# Dental caries and erosion status of 12-year-old Hong Kong children

**DOI:** 10.1186/1471-2458-14-7

**Published:** 2014-01-08

**Authors:** Shinan Zhang, Alex MH Chau, Edward CM Lo, Chun-Hung Chu

**Affiliations:** 1 Faculty of Dentistry, The University of Hong Kong, 34 Hospital Road, Hong Kong, SAR, China

**Keywords:** Caries, Dental erosion, Oral health behaviours, Epidemiology, Children

## Abstract

**Background:**

This study aimed to assess the dental caries and erosion status of 12-year-old Hong Kong children and study the determinants of dental caries and dental erosion of these children.

**Methods:**

The survey was performed from 2011 to 2012 with ethics approval. Stratified random sampling was adopted to select 12-year-old children in 7 primary schools in Hong Kong. The participating parents were asked to complete a self-administered questionnaire concerning their children’s diet and oral health habits. The children were examined for caries status with WHO criteria by 3 calibrated examiners. Detection of dental erosion followed Basic Erosive Wear Examination (BEWE) criteria.

**Results:**

A total of 704 children were recruited and 600 (316 boys, 53%) participated in the survey. There were 124 children (21%) with caries experience (DMFT > 0) and their DMFT was 0.34 ± 0.76. About half of their decay was unfilled (DT = 0.16 ± 0.52) The DMFT of girls and boys were 0.45 ± 0.89 and 0.23 ± 0.61, respectively (p = 0.001). Girls also had a higher DT (0.21 ± 0.62 compared with 0.11 ± 0.41, p = 0.013) and FT than boys (0.23 ± 0.63 compared with 0.12 ± 0.44, p = 0.016). Most children (75%) had at least some sign of erosion (BEWE > 0), but no severe erosion (BEWE = 3). Logistic regression showed girls who consumed soft drinks and took vitamin C supplements had higher caries risk. Dental erosion was more severe among the children who had caries experience and consumed fruit juice.

**Conclusions:**

The 12-year-old Hong Kong children had low caries experience, and almost half of the decay was left untreated. Although severe erosion was not found, many children had early signs of erosion.

## Background

Although the dental caries in children has generally declined in the past 50 years [1], dental caries of children is still prevalent in many countries and disadvantaged communities and is regarded as global major public health problem [2,3]. Bagramian et al. performed a review of epidemiological data and found that caries prevalence among children has increased in many developed and developing countries in the past 10 years [4]. Oral health is an essential part of general health [5]. Tooth decay, if left untreated, will progress and infect the dental pulp, causing pain and forming dental abscess. Moreover, it can lead to severe local and systemic infections [6]. This increase in dental caries signals a pending public health crisis to many communities.

Water fluoridation was implemented in Hong Kong in 1961 and the caries experience of children has reduced [7]. An oral health education unit has been established to promote dental health and good oral health-related behaviours in the community. In addition, the School Dental Care Service (SCDS) was established in 1980 to provide basic curative treatments for children attending primary schools in Hong Kong [8]. There is, however, no continuation of dental care when the children enter secondary school, and they have to seek private dentists for check-ups and dental care.

Dental erosion is tooth surface loss caused by chemical processes without bacterial involvement [9]. The impacts of dental erosion include compromised aesthetics, dentinal hypersensitivity, and reduced chewing ability, all of which may lead to complicated treatment and high treatment costs if advanced erosion occurs [10]. Dental treatment for dental erosion can be very complex and challenging and early diagnosis is therefore important. However, many child patients are not aware of the problem and clinicians may sometimes overlook or fail to detect early stage erosion because the patient often does not present symptoms [11]. Dental erosion is becoming a public health concern because of its high prevalence in population surveys [12,13]. Newly erupted permanent teeth of children have immature enamel, which is more susceptible to acid attack by soft drinks. Certain drinks, such as sport/energy drinks, ‘healthy’ drinks, and soda, are popular among them [14] but are also acidic and have fermentable sugars [15]. Therefore, it is plausible that more children will be affected by dental erosion [10]. A survey in Iceland reported 31% of 15-year-old teenagers had signs of erosion, and 5.5% of children had severe erosion on their teeth [12]. Another study in Sweden found that 34% of 18–19 year old adults had at least one tooth with severe erosion extending to dentine [13].

The Department of Health performed a population-based oral health survey among 5- and 12-year-old children in 2001 [16]. The survey followed the World Health Organization (WHO) guidelines on dental caries assessment [17], but there is no report on dental erosion. Currently, the literature on the distribution and epidemiology of dental erosion among Chinese young adults is scarce. A literature search found no related studies reported in English so far. In order to better understand the dental health of children in Hong Kong, an updated survey on a representative sample is warranted. The collected information would be useful to the dental profession and the government for planning dental services in Hong Kong. The objectives of this study were to first investigate dental caries, prevalence, and the distribution of dental erosion in 12-year-old children in Hong Kong and second, to investigate the risk factors for dental erosion and dental caries.

## Methods

### Selection of children

This study was approved by the Institutional Review Board of the University of Hong Kong/Hospital Authority Hong Kong West Cluster (IRB UW11-169). It was carried out from 2011 to 2012. During this time, there were approximately 66,000 12-year-old children living in Hong Kong [18]. They lived in 3 main geographical areas, namely Hong Kong Island (10,000 children), Kowloon (19,000 children), and the New Territories (37,000 children). Approximately 700 12-year-old children were recruited after the sample size determination. Stratified cluster sampling was employed to select 7 primary schools from the list of primary schools according to the population in the 3 geographical areas. Since each primary school had more or less 100 12-year-old students, this study randomly selected 1 school in Hong Kong Island, 2 from Kowloon and 4 from the New Territory. All 7 schools selected agreed to participate in this study. Oral health education was given to the school children prior to the survey. All children who were 12 years old were invited to participate in this study. A letter was sent to the children’s parents informing them of the study and to ask for their consent. Children with serious general health problems, such as congenital heart disease, were excluded from the study.

### Sample size estimation

The sample size calculation was performed with reference to a previous survey of school children in Hong Kong which reported a mean DMFT score of 0.78 ± 1.19 [19]. The 95% confidence interval was set to be 0.1 on both sides of the mean DMFT score. The computed minimum sample size was 547. Assuming a response rate of 80%, the number of preschool children to be invited would be at least 684.

### Questionnaire survey

A visit was paid to each primary school before the survey to deliver the questionnaires and to discuss the protocol with the primary school teachers. The parents were asked to complete a self-administered questionnaire (attached as Additional file 1). The questionnaire consisted of four parts and the following information was collected:

1) The child’s personal data: sex, age, place of birth.

2) The child’s frequency of toothbrushing.

3) The child’s dietary habit: frequency of acidic drinks and fruit juice, taking vitamin C supplement drinks, and use of chewing gum.

4) Parent’s dental knowledge: To assess the parents’ dental knowledge, there were 21 multiple choice questions in the questionnaire on the causes and prevention of dental diseases [7]. One score was given to each correct answer, and no score was given to a wrong or ‘don’t know’ answer. Thus, the total dental knowledge score could range from 0 to 21. The parents were then categorized into three groups according to their dental knowledge scores in 3 equal intervals: low (scored 0–7), middle (scored 8–14), and high (scored 15–21).

### Clinical examination

The clinical examinations were carried out by 3 trained and calibrated dentists using a disposable front surface dental mirror, intra-oral LED light, and a ball-ended WHO CPI probe. A diagnosis of dental caries of permanent teeth was made according to the criteria recommended by the World Health Organization [17]. Caries was charted when a lesion in a pit or fissure, or on a smooth tooth surface, had an unmistakable cavity, undermined enamel, or a detectably softened floor or wall. The DMFT index was used to record the dental caries experience of the permanent dentition of the children. Calibration of examiners with an experienced oral epidemiologist was carried out one week prior to the survey. Duplicate examinations were carried out on 10% of the children during the survey. A Kappa statistic was used to assess the inter-examiner reproducibility.

The BEWE criteria [20] were adopted to assess dental erosion status. The buccal, lingual, and occlusal/incisal surfaces of all teeth were examined. The surface with the highest-coded BEWE score in a sextant was recorded to represent the whole sextant. One-tenth of the participants were systematically re-examined to assess and monitor the inter-examiner agreement. The participants were notified about their dental status (presence of tooth decay) after the examination. Those who had dental caries were suggested to have an appointment with the SCDS or their own dentist for follow-up treatment at cost. A calibration exercise was performed on a convenience sample of thirty-six 11- to 12-year-old primary school children 1 week before the survey. The 3 dentists examined each child in turn. The dentists would re-examine the child when there was disagreement on the diagnosis. The findings of each assessment were discussed until agreement was achieved.

### Statistical analysis

Data analysis was performed using the software Statistical Package for Social Sciences, version 17.0 (SPSS Inc., Chicago, Illinois, USA). Independent t-test and one-way ANOVA were used to assess the statistical significance of the differences in the studied continuous variables between the groups. Multi-factor ANOVA was used to investigate the effects of the independent variables studied, including the demographic and socio-economic background of the child and the child’s oral health-related behaviours, on the child’s dental erosion status. Logistic regression was used to study the relationship between the child’s dental caries prevalence and the independent variables. A backward stepwise procedure was used to remove the variables that were not statistically significant. The final model only contained variables that were statistically significant. The level of statistical significance for all tests was set at 0.05.

## Results

A total of 704 children in 7 primary schools were invited to participate in this study. Of those, 104 failed to complete the consent form, questionnaire, or the oral examination procedure due to uncooperative behaviour or absence from school. Therefore, 600 children were examined. The response rate was 85% (600/704). Among the 600 children examined, 60 were in Hong Kong, 234 were in Kowloon, and 306 were in New Territory, and approximately half (n = 316, 53%) of them were boys (Table 1). Kappa values for assessment of caries status and dental erosion were 0.95 to 0.97 (0.95, 0.97, 0.97) and 0.75 to 0.80 (0.75, 0.77, 0.80), respectively.

**Table 1:** Caries status according to variables studied

**Variable (N)**	**DMFT > 0 (n, %)**	**p-value**
Children examined (600)	21%	
Gender (600)		
Girls (284)	73, 26%	0.004
Boys (316)	51,16%	
Place of birth (577)		
Hong Kong (491)	95, 19%	0.185
Not in Hong Kong (86)	22, 26%	
Frequency of soft drinks (600)		
At least once every two days (120)	34, 28%	0.020
Less than once every two days (480)	90, 19%	
Frequency of citric tea/drinks containing lemon (600)		
At least once every two days (113)	19, 17%	0.262
Less than once every two days (487)	105, 22%	
Frequency of fruit juice (600)		
At least once every two days (115)	27, 24%	0.408
Less than once every two days (485)	97, 20%	
Frequency of chewing gum (600)		
At least once every two days (70)	15, 21%	0.867
Less than once every two days (530)	109, 21%	
Frequency of Vitamin C supplement drinks (600)		
At least once every two days (82)	29, 35%	<0.001
Less than once every two days (518)	95, 18%	
Frequency of toothbrushing (600)		
At least once a day (144)	34, 24%	0.317
Twice or more (456)	90, 20%	
Care taker (598)		
Parents (501)	109, 22%	0.162
Others (97)	15, 16%	
Education of father (573)		
Primary or below (61)	16, 26%	0.579
Secondary (395)	83, 21%	
Tertiary or above (117)	23, 20%	
Education of mother (585)		
Primary or below (64)	15, 23%	0.848
Secondary (434)	89, 21%	
Tertiary or above (87)	19, 22%	
Parent’ dental knowledge (600)		
Low (53)	9, 17%	0.534
Middle (534)	111, 21%	
High (13)	4, 31%	
Erosion experience (600)		
No (150)	27, 18%	0.352
Yes (450)	97, 22%	

### Dental caries status

The caries experience of the children in DMFT (±SD) was 0.34 ± 0.76. Approximately half of the score was contributed by untreated decayed teeth (mean DT = 0.16 ± 0.52). Girls had a higher DMFT than boys (0.45 ± 0.89 compared with 0.23 ± 0.61, p = 0.001). Girls also had more untreated decay teeth (DT) than boys (0.21 ± 0.62 compared with 0.11 ± 0.41, p = 0.013). The proportion of children with dental caries experience (DMFT > 0) was 21% (n = 124). Out of these 124 children, only 1% (n = 14) had DMFT equal to or higher than 3. Among all the children examined, only 1 missing tooth was found due to caries. Results of binary logistic regression showed that the prevalence of dental caries were significantly related to the habit of drinking soft drinks (OR: 1.952, 95% C.I: 1.193-3.194) and taking vitamin C supplements (OR: 2.157, 95% C.I: 1.301-3.172) (Table 2). The odds for girls having caries experience (DMFT > 0) were 1.751 times more than for boys. There is no collinearity of the frequencies of consuming soft drinks, fruit juice and vitamin C supplement because they were not significantly inter-correlated, adjusted for prevalence of dental caries. The final model obtained by backward stepwise binary regression was the same as that obtained from the forward stepwise procedure.

**Table 2:** Relationship between caries prevalence (DMFT > 0) and variables selected

**Factor**	**DMFT > 0 (n, %)**	**Odds ratio**	**95% C.I.**	**p-value**
Frequency of soft drinks				
At least once every 2 days	34, 28%	1.952	1.193 - 3.194	0.030
Less than once every 2 days^#^	90, 19%			
Frequency of Vitamin C supplement drinks				
At least once every 2 days	29, 35%	2.157	1.301 - 3.172	0.003
Less than once every 2 days^#^	95, 18%			
Gender				
Girls	73, 26%	1.751	1.141 - 2.687	0.010
Boys^#^	51, 16%			
(Constant)		0.149		<0.001

^#^Reference group.

### Dental erosion status

A quarter (25%, n = 150) of the children were found to be free from dental erosion (BEWE score = 6 for all 6 sextants). None of the children were found to have severe erosion (BEWE score = 3), and 9 (1.5%) of the children had teeth in 1 or 2 sextants with distinctive erosion (BEWE score = 2). The sum of the scores of the 6 sextants in each child ranged from 0 to 13, and the mean was 1.7 ± 1.6 (Table 3). The eroded sextant in each child ranged from 0 to 6, and the mean number of eroded sextants in each child was 1.6 ± 1.4. Gender and place of birth were statistically not associated with the BEWE scores (p > 0.05) (Table 3). However, children who took fruit juice at least once every 2 days and children with caries experience had significantly higher BEWE scores (Table 4).

**Table 3:** Erosion and variables studied

**Variable (N)**	**BEWE (±SD)**	**p-value**
Children examined (600)	1.7 ± 1.6	
Gender (600)		
Girls (284)	1.8 ± 1.8	0.166
Boys (316)	1.6 ± 1.4	
Place of birth (577)		
Hong Kong (491)	1.7 ± 1.6	0.438
Not in Hong Kong (86)	1.8 ± 1.7	
Frequency of soft drinks (600)		
At least once every two days (120)	1.9 ± 2.1	0.110
Less than once every two days (480)	1.6 ± 1.4	
Frequency of citric tea / drinks containing lemon (600)		
At least once every two days (113)	1.8 ± 1.7	0.194
Less than once every two days (487)	1.6 ± 1.5	
Frequency of fruit juice (600)		
At least once every two days (115)	2.0 ± 1.7	0.015
Less than once every two days (485)	1.6 ± 1.5	
Frequency of chewing gum (600)		
At least once every two days (70)	2.0 ± 2.1	0.046
Less than once every two days (530)	1.6 ± 1.5	
Frequency of Vitamin C supplement drinks (600)		
At least once every two days (82)	2.0 ± 2.0	0.064
Less than once every two days (518)	1.6 ± 1.4	
Frequency of toothbrushing (600)		
At least once a day (144)	2.0 ± 1.9	0.017
Twice or more (456)	1.6 ± 1.5	
Care taker (598)		
Parents (501)	1.6 ± 1.6	0.233
Others (97)	1.8 ± 1.6	
Education of father (573)		
Primary or below (61)	1.9 ± 1.8	0.574
Secondary (395)	1.6 ± 1.7	
Tertiary or above (117)	1.7 ± 1.2	
Education of mother (585)		
Primary or below (64)	1.8 ± 1.7	0.614
Secondary (434)	1.7 ± 1.6	
Tertiary or above (87)	1.2 ± 1.1	
Parent’ dental knowledge (600)		
Low (53)	1.7 ± 1.5	0.665
Middle (534)	1.7 ± 1.6	
High (13)	1.3 ± 2.0	
Caries experience (600)		
No (476)	1.5 ± 1.4	0.004
Yes (124)	2.1 ± 2.1	

**Table 4:** Relationship between BEWE scores and the selected variables

**Factor**	**Beta**	**SE**	**p-value**
Frequency of fruit juice			
At least once every 2 days	0.377	0.163	0.021
Less than once every 2 days^#^			
Caries experience			
Yes	0.591	0.161	<0.001
No^#^			
(Constant)	1.470	0.078	<0.001

R^2^ = 0.032 (Adjusted R^2^ = 0.029); ^#^Reference group.

## Discussion

A stratified cluster sampling technique was employed in this study. It is important to note that cluster sampling can increase the sampling error because children in the same strata can be similar and are less likely to be representative of the population. However, this technique is an efficient sampling method to obtain a representative sample from a population with non-overlapping classifications, and the factor classifying the population may be significant on the study parameter. In this study, 3 geographical regions in Hong Kong were chosen as the strata because the household income of the families living in different regions may be different [21], and household income was found to be a significant factor on caries experience and dental erosion in some studies [7,22,23]. Therefore, questions on household income, considered to be sensitive in Hong Kong culture, could be omitted. Apart from avoiding sensitive questions, oral health seminars on preventing caries and erosion were given to the children after the oral examination, which was welcomed by the schools as part of their health education in their regular syllabi. This could explain the high response rate of 85%.

Two common assessment methods for dental erosion were Visual Erosion Dental Examination (VEDE) [24] and Basic Erosive Wear Examination (BEWE) [25]. Studies reported that the intra- and inter-examiner agreement in both systems had acceptable levels of reliability [20,24]. The BEWE system was developed in 2007 following an international workshop on erosion indices [20]. It was recognized that previous indices lacked validity, as they mostly measured general tooth wearing instead of erosive wear [25]; still, there was no intention to distinguish erosive wear from other forms of wear in the BEWE system [20]. The use of the BEWE diagnostic criteria and recording system is not free from error. Therefore, it is essential to point out that the wear examined in this study is not necessarily due to dental erosion. In addition, it does not differentiate between enamel and dentine because surfaces are scored irrespective of structure. Diagnosis of early erosive wear can be difficult, and the extent of wearing by attrition and abrasion can also be difficult to determine [20].

Re-examination of the students was performed on the same day to minimize disturbance to the school. The 3 examiners examined about 85 students each day at a given school. Each examiner would examine about 28 students and were likely to remember the tooth statuses of the re-examined children. Since the examiners were trained and their intra-examiner agreements were high in the calibration exercise, intra-examiner agreement was not assessed in the main study. Moreover, there would be many re-examined children if intra- and inter-examinations for all 3 examiners were conducted. This study found a reasonable inter-examiner agreement on the BEWE score between the examiners. Another study reported that BEWE was a convenient index with adequate sensitivity and specificity, but possibly not sensitive enough to detect the severity of some erosive lesions [26]. Currently, there is no consensus regarding which index possesses absolute superiority over any others. BEWE was found to be a convenient index. The tooth with highest BEWE score (or the most severely affected tooth) in a sextant was recorded. The examiners in this study spent approximately 2 minutes on each child for erosion assessment. However, it is important to note that the 6 upper anterior teeth can be all eroded with no other teeth affected, and this is recorded as 1 sextant having erosion; however, 2 sextants having erosion are recorded if only 2 teeth in 2 sextants are eroded. The count of the number of eroded teeth, rather than of the sextant, is more precise but takes more time in the clinical assessment. Measuring the highest BEWE score of the sextant rather than the number of eroded teeth is quick and simple to perform, but the results collected lack discriminatory power. In this study, only a few children had teeth with distinctive erosion (BEWE score = 2) and no children had a BEWE score equal to 3, reflecting that the cut-off points of the grading in BEWE might be too high in this sample. An index with lower threshold values may be more suitable in our sample [27].

This study found that the caries experience in mean DMFT score dropped from 0.8 in 2001, to 0.3 in 2012. This may be because of water fluoridation at 0.5 ppm since 1988, as well as the comprehensive preventive dental services provided by the SDCS [28]. All children enrolled in local primary schools in Hong Kong were entitled to receive oral examination and treatment by the SDCS with an annual fee of approximately US $3 paid by the parents or guardians of the child. The treatment was provided by dental therapists and included fluoride gel application, fissure sealants, restorations, and extraction. In our study, a large proportion of the children had fissure sealants on their molars, which was probably performed by the SDCS dental therapists. The current mean DMFT score in Hong Kong was also low when compared to nearby countries such as Cambodia [29], China [30], and Thailand [31]. However, these studies had identified various associated factors on caries experience. Since water fluoridation and dental visit patterns in our samples were different from those in other countries, caution should be taken when oral health data are compared internationally.

The prevalence of dental erosion was higher in this study when compared to another study conducted earlier in southern China [32]. The difference may be attributed to geographical and socioeconomic factors. In our study, the survey was performed in Hong Kong, a well-developed city where acidic food and beverages are easily available. Many people in Hong Kong frequently drink fruit juices, a habit that is considered as part of a healthy diet. In this study, frequent fruit juice consumption increased tooth wear, and this result disagreed with the study by Wang et al. [31]. One should be cautious when interpreting this result, as a wide spectrum of erosive power existed within each category of beverages [33]. This study also found children who had caries experience had higher BEWE scores than those who had no caries experience. It was plausible that children who had caries consumed more acidic food. Abu-Ghazaleh et al. [34] reported increased prevalence of wear in males compared to females, but this study found no statistically significant association of erosion with gender.

The closed-ended questionnaire used in this study aimed for an overview of fruit juice consumption. Information such as the kind of fruit juices, acidity, and scale of frequency of consumption were not recorded. Some recent epidemiological studies on dental erosion did not consider the dependency and interaction terms among the factors investigated [31,35,36]. Because of the complex interplay among the risk factors on dental erosion, qualitative exploratory studies on biological, dietary, lifestyle, and cultural factors are necessary. Moreover, a more in-depth analysis on the interaction among these factors could be carried out.

## Conclusion

In this study, about one-fifth of 12-year-old children had caries experience and about half of the decay was left untreated. High frequency of soft drinks, vitamin C supplements and being a girl were found to be positively associated with caries prevalence. Many children had early signs of erosion, which was related to fruit juice consumption. Screening programmes can be provided to identify risk groups for early preventive measures. This can strategically enhance the cost-effectiveness of dental treatment.

## Competing interests

The authors declare that they have no competing interests.

## Authors’ contributions

SN and AMH: performed the dental examination, data entry, and analysis and prepared the manuscript. CH: performed the dental examination and supervised fieldworkers during the survey. ECM: provided training, checked data analysis, and revised the manuscript. All authors read and approved the final manuscript.

## Pre-publication history

The pre-publication history for this paper can be accessed here:

http://www.biomedcentral.com/1471-2458/14/7/prepub

## Supplementary Material

Additional file 1 Oral health questionnaire. Click here for file

## References

[B1] Reich E Trends in caries and periodontal health epidemiology in Europe Int Dent J 2011 51 S6 392 398 11794560 10.1111/j.1875-595x.2001.tb00585.x

[B2] Petersen PE The world oral health report 2003: continuous improvement of oral health in the 21st century – the approach of the WHO global oral health programme Community Dental Oral Epidemiol 2003 31 s1 3 24 10.1046/j..2003.com122.x 15015736

[B3] Petersen PE Lennon MA Effective use of fluorides for the prevention of dental caries in the 21st century: the WHO approach Community Dental Oral Epidemiol 2004 32 5 319 321 10.1111/j.1600-0528.2004.00175.x 15341615

[B4] Bagramian RA Garcia-Godoy F Volpe AR The global increase in dental caries: a pending public health crisis Am J Dent 2009 22 3 8 19281105

[B5] Chu CH Chau AM Lo EC Lam A Planning and implementation of community oral health programs for caries management in children Gen Dent 2012 60 210 215 22623460

[B6] Chu CH Treatment of early childhood caries: a review and case report Gen Dent 2000 48 2 42 48 11199573

[B7] Chu CH Fung DSH Lo ECM Dental caries status of pre-school children in Hong Kong Br Dent J 1999 187 11 616 620 16163284 10.1038/sj.bdj.4800347

[B8] Chu CH Wong SSS Suen RPC Lo ECM Oral health and dental care in Hong Kong Surgeon 2013 11 3 153 157 10.1016/j.surge.2012.12.010 23507329

[B9] Imfeld T Dental erosion: definition, classification and links Eur J Oral Sci 1996 104 2 151 155 10.1111/j.1600-0722.1996.tb00063.x 8804882

[B10] Chu CH Lam A Lo ECM Dentin hypersensitivity and its management Gen Dent 2011 59 2 115 122 21903521

[B11] Lussi A Schaffner M Jaeggi T Dental erosion - diagnosis and prevention in children and adults Int Dent J 2007 57 385 398

[B12] Arnadottir IB Holbrook WP Eggertsson H Gudmundsdottir H Jonsson SH Gudlaugsson JO Saemundsson SR Eliasson ST Agustsdottir H Prevalence of dental erosion in children: a national survey Community Dent Oral Epidemiol 2010 38 6 521 526 10.1111/j.1600-0528.2010.00559.x 20690934

[B13] Hasselkvist A Johansson A Johansson AK Dental erosion and soft drink consumption in Swedish children and adolescents and the development of a simplified erosion partial recording system Swed Dent J 2010 34 4 187 195 21306084

[B14] Chu CH Pang KKL Lo ECM Dietary behaviour and knowledge of dental erosion among Chinese adults BMC Oral Health 2010 10 13 10.1186/1472-6831-10-13 20525244 PMC2894740

[B15] Owens BM The potential effects of pH and buffering capacity on dental erosion Gen Dent 2007 55 6 527 531 18050578

[B16] Department of Health Oral health survey 2001 2002 Hong Kong: Hong Kong SAR Government

[B17] World Health Organization Oral healthy surveys: basic methods 1997 4 Geneva: World Health Organization

[B18] Census and Statistics Department 2011 Population census: http://www.census2011.gov.hk/en/index.html Access on November 19, 2013

[B19] Department of Health Oral health survey 2001 technical report 2002 Hong Kong: Department of Health

[B20] Bartlett D Ganss C Lussi A Basic erosive wear examination (BEWE): a new scoring system for scientific and clinical needs Clin Oral Investig 2008 12 1 S65 S68 18228057 10.1007/s00784-007-0181-5 PMC2238785

[B21] Census and Statistics Department Thematic report household income distribution in Hong Kong published in June 2012: http://www.census2011.gov.hk/pdf/household-income.pdf Accessed on April 4, 2013

[B22] Harding MA Whelton H O’Mullane DM Cronin M Dental erosion in 5-year-old Irish school children and associated factors: a pilot study Community Dent Health 2006 20 3 165 170 12940307

[B23] Matonanaki M Koletsi-Kounari H Mamai-Homata E Papaioannou W Dental erosion prevalence and associated risk indicators among preschool children in Athens, Greece Clin Oral Investig 2013 17 2 585 593 10.1007/s00784-012-0730-4 22526894

[B24] Mulic A Tveit AB Wang NJ Hove LH Espelid I Skaare AB Reliability of two clinical scoring systems for dental erosive wear Caries Res 2010 44 3 294 299 10.1159/000314811 20516691

[B25] Ganss C How valid are current diagnostic criteria for dental erosion? Clin Oral Investig 2008 12 S41 S49 10.1007/s00784-007-0175-3 18228062 PMC2238791

[B26] Margaritis V Mamai-Homata E Koletsi-Kounari H Polychronopoulou A Evaluation of three different scoring systems for dental erosion: a comparative study in adolescents J Dent 2011 39 1 88 93 10.1016/j.jdent.2010.10.014 21035516

[B27] Milosevic A The problem with an epidemiological index for dental erosion Br Dent J 2011 211 5 201 203 10.1038/sj.bdj.2011.722 21904350

[B28] Chu CH Ho PL Lo EC Oral health status and behaviours of preschool children in Hong Kong BMC Public Health 2012 12 767 10.1186/1471-2458-12-767 22966820 PMC3490858

[B29] Chu CH AWYW Lo ECM Courtel F Oral health status and behaviours of children in rural districts of Cambodia Int Dent J 2008 58 15 22 18350849 10.1111/j.1875-595x.2008.tb00172.x

[B30] Wong MC Lo ECM Schwarz E Zhang HG Oral health status and oral health behaviors in Chinese children J Dent Res 2001 80 5 1459 1465 10.1177/00220345010800051501 11437220

[B31] Narksawat K Tonmukayakul U Boonthum A Association between nutritional status and dental caries in permanent dentition among primary schoolchildren aged 12–14 years, Thailand Southeast Asian J Trop Med Public Health 2009 40 2 338 344 19323020

[B32] Wang P Lin HC Chen JH Liang HY The prevalence of dental erosion and associated risk factors in 12-13-year-old school children in Southern China BMC Public Health 2010 10 478 10.1186/1471-2458-10-478 20704718 PMC2927543

[B33] Lussi A Jaeggi T Zero D The role of diet in the aetiology of dental erosion Caries Res 2004 38 1 34 44 10.1159/000073918 14685022

[B34] Abu-Ghazaleh SB Burnside G Milosevic A The prevalence and associated risk factors for tooth wear and dental erosion in 15- to 16-year-old schoolchildren in Amman, Jordan Eur Arch Paediatr Dent 2013 14 21 27 10.1007/s40368-012-0006-3 23532810

[B35] Huew R Waterhouse P Moynihan P Kometa S Maguire A Dental caries and its association with diet and dental erosion in Libyan schoolchildren Int J Paediatr Dent 2012 22 1 68 76 10.1111/j.1365-263X.2011.01170.x 21831127

[B36] Hamasha AA Zawaideh FI Al-Hadithy RT Risk indicators associated with dental erosion among Jordanian school children aged 12–14 years of age Int J Paediatr Dent 2013 24 56 58 23432693 10.1111/ipd.12026

