# Supplementary material for: Dental caries and erosion status of 12-year-old Hong Kong children
Source: BMC Public Health. 2014 Jan 8;14:7. doi: 10.1186/1471-2458-14-7 (PMC3890525; doi:10.1186/1471-2458-14-7)
Supplement: Additional file 1 — Oral health questionnaire. [file 1471-2458-14-7-S1.docx]

Oral Health Questionnaire

Part A: Dietary habit of your child

1. How often does your child have soft drinks?

□ 6, 7 times a week or more (almost every day or more)

□ 3 times a week (alternate days)

□ Once a week

□ Less than once a week / never

1. How often does your child have citric tea / drinks containing lemon?

□ 6, 7 times a week or more (almost every day or more)

□ 3 times a week (alternate days)

□ Once a week

□ Less than once a week / never

1. How often does your child drink fruit juice?

□ 6, 7 times a week or more (almost every day or more)

□ 3 times a week (alternate days)

□ Once a week

□ Less than once a week / never

1. How often does your child have chewing gum?

□ 6, 7 times a week or more (almost every day or more)

□ 3 times a week (alternate days)

□ Once a week

□ Less than once a week / never

1. How often does your child have vitamin C supplement drinks?

□ 6, 7 times a week or more (almost every day or more)

□ 3 times a week (alternate days)

□ Once a week

□ Less than once a week / never

Part B: Toothbrushing habit

1. How often does your child brush his/her teeth?

□ Never / irregularly

□ Once a day

□ Twice a day

□ Three times a day or more

Part C: Parent’s dental knowledge

7). The causes of dental decay include:

|  | Yes | No | Don’t know |
| --- | --- | --- | --- |
| a) Too much consumption of candies | □ | □ | □ |
| b) Unclean teeth | □ | □ | □ |
| c) Tooth worms attack | □ | □ | □ |
| d) “Hot air” | □ | □ | □ |

8). Preventions of tooth decay include:

|  | Yes | No | Don’t know |
| --- | --- | --- | --- |
| a) Medicine | □ | □ | □ |
| b) Herbal tea | □ | □ | □ |
| c) Use of fluoridated toothpaste | □ | □ | □ |
| d) Decrease frequency of sugar consumption | □ | □ | □ |

9). Effects of fluoride to teeth include:

|  | Yes | No | Don’t know |
| --- | --- | --- | --- |
| a) No effect | □ | □ | □ |
| b) Prevent tooth decay | □ | □ | □ |
| c) Tooth whitening | □ | □ | □ |
| d) Prevent periodontal disease | □ | □ | □ |

10). Which of the following food can cause tooth decay?

|  | Yes | No | Don’t know |
| --- | --- | --- | --- |
| a) Soft drinks | □ | □ | □ |
| b) Ice-cream | □ | □ | □ |
| c) Cheese | □ | □ | □ |
| d) Peanuts | □ | □ | □ |

11). The causes of gum bleeding include:

|  | Yes | No | Don’t know |
| --- | --- | --- | --- |
| a) Unclean teeth | □ | □ | □ |
| b) It Is a normal phenomenon | □ | □ | □ |

12). Methods to prevent periodontal disease include:

|  | Yes | No | Don’t know |
| --- | --- | --- | --- |
| a) Tooth brushing | □ | □ | □ |
| b) Saline mouth-rinsing | □ | □ | □ |
| c) Regular scaling (professional tooth cleaning) | □ | □ | □ |

Part D: Others

13). Who is the main carer of the child? (Please choose ONLY ONE OPTION from below)

□ Parents

□ Grandparents

□ Other relatives

□ Domestic helper

□ Friends / neighbours / others

14). The educational level of the parents who live with the child:

Father Mother

Primary or below □ □

Secondary □ □

Tertiary or above □ □

Part E: Information of child

Child name: Class:

Gender: □Male □Female

Date of Birth (DD-MM-YYYY):

Place of Birth: □ Hong Kong

□ Mainland China

□ Others (please specify):

Contact Number (for further follow-up):

- Thank you -
